# Listening to a Three‐Dimensional Virtual Reality Sound: Effects on Stress Response of Adults

**DOI:** 10.1002/brb3.70418

**Published:** 2025-03-18

**Authors:** Yücehan Yilmaz, Faruk Dişli, Sedat Yildiz

**Affiliations:** ^1^ Department of Physiology, Faculty of Medicine University of Adiyaman Adiyaman Türkiye; ^2^ Department of Physiology, Faculty of Medicine University of Inonu Malatya Türkiye

**Keywords:** 3D sound, autonomous nervous system, HRV, hypothalamo–pituitary–adrenal axis, salivary cortisol, virtual reality

## Abstract

**Introduction:**

Virtual reality (VR) technologies utilizing three‐dimensional (3D) sound may offer sensually engaging imitations. Thus, they may lead to relaxation or may provide a way of escaping from disturbing life events. As they have the potential to manage stress, the current study aimed to investigate the effects of VR 3D sounds on the stress axes of the body, namely the autonomic nervous system (ANS) and hypothalamo–pituitary–adrenal (HPA).

**Methods:**

Participants (19 men and 27 women, mean age 25.8 ± 10.4 years), wearing on‐ear headphones, did not listen to anything (control) or listened to a mono or 3D sound imitating a real‐life situation taking place in a virtual barbershop. The control phase was immediately followed by mono or 3D phases. Half of the participants in mono and 3D phases were crossed, with 45 min intervals, to control the effect of time and habituation. Reactivity of ANS and HPA was measured by heart rate variability (HRV) and salivary cortisol measurements, respectively. HRV was measured during each phase by a 5‐min continuous electrocardiogram recording. Salivary samples were taken during control recording and 15‐min after mono or 3D sound listening. HRV parameters consisted of both time‐ and frequency‐domain parameters, including standard deviation of R–R interval (SDNN), total power (TP), and low frequency/high frequency (LF/HF), as a measure for sympathovagal activity.

**Results:**

Compared to the control phase, the 3D sound increased almost all HRV parameters (including SDNN, TP, LF/HF, etc., *p* < 0.05) but did not affect salivary cortisol levels (*p* > 0.05). Effects of mono sound were in between the control and 3D sound phases.

**Conclusion:**

It seems that a 3D sound imitating a real‐life environment does not affect HPA but increases HRV and sympathovagal balance, suggesting that VR 3D sound is likely to reproduce an ANS response observed in real life.

## Graphical abstract

It seems that a 3D sound imitating a real life environment does not affect hypothalamo–pituitary–adrenal axis but increases heart rate variability and sympathovagal balance, suggesting that virtual reality 3D sound is likely to reproduce an autonomic nervous system response observed in real life.

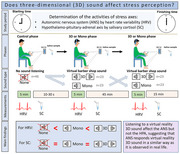

## 1 Introduction

The evolution of sound technology has profoundly impacted human life. Earlier, the sound was limited to mono audio transmitted through a single channel. With advancements in technology, new solutions were sought to enable sound to be experienced in a much richer and more realistic manner, leading to the advent of stereo sound systems and the subsequent development of three‐dimensional (3D) sound technologies (Wycisk et al. 2022). 3D sound provided a multidimensional perception that incorporates the direction, height, and depth of sound, allowing us to experience the source of sound in a more realistic way (Blommer and Greenberg 2003; Brinkman et al. 2015; Kim et al. 2019).

The shift from mono to stereo to 3D has revolutionized many fields, including entertainment sectors like music, cinema, gaming, etc. (André et al. 2012). Many people spend several hours on a weekly or daily basis on 3D sound‐requiring activities (Ma et al. 2020), and this has been boosted during the post‐pandemic era (Hurst 2020). In the areas of stress management and mental health, the use of sound‐based methods has also grown in importance as a noninvasive solution to common health issues (Latif et al. 2015). By immersing users in emotionally engaging environments, 3D sounds created by VR technology may promote calming effects and relaxation and consequently may have a high potential to decrease stress levels in the body (Meshkat et al. 2024).

Stress is a complex process that exerts widespread effects on various biological systems in the body. There are two main systems governing the effects of stress, namely the autonomic nervous system (ANS) and hypothalamic–pituitary–adrenal (HPA) axis. As stress increases, the ANS undergoes changes, with the sympathetic nervous system being activated, whereas parasympathetic activity being depressed. Similarly, as stress persists, the HPA axis is activated, leading to an increase in cortisol secretion (Guilliams and Edwards 2010). In this context, both ANS and HPA activity can be used together to monitor stress responses (Ucar et al. 2021). ANS activity can be determined by using heart rate variability (HRV) to assess sympathovagal balance, whereas HPA activity can be determined by measuring salivary cortisol concentrations (Ozgocer, Yildiz, et al. 2017; Rajendra Acharya et al. 2006). Due to its sensitivity to psychological factors such as stress and relaxation, both salivary cortisol and HRV are commonly used in physiological, psychological, and neuroendocrinological studies (Thayer et al. 2012; Erbay et al. 2018; Ozgocer, Ucar, et al. 2017; Ucar et al. 2021).

Although the use of 3D audio technology for entertainment purposes has become widespread among individuals, its effects on both stress and stress axes have not been well elucidated. In this context, we hypothesized that a VR sound imitating a 3D environment will cause relaxation and decrease the activity of both stress axes. To test this hypothesis, we investigated the effects of mono and 3D sounds on the activities of ANS and HPA by measuring salivary cortisol and HRV, respectively.

## 2 Methods

### 2.1 Participants

The participants consisted of 46 people, including 19 males and 27 females. The participants had an average age of 25.82 ± 10.37 years. Most of them were university students. The participants were healthy and had no chronic diseases. At each stage, 5 min of HRV was recorded. The participants whose rhythm disturbance and extrasystole were found during HRV recording were excluded from the study. The present study was approved by the Clinical Trials Ethics Committee of the “Inonu University” (No: 2022/68). The participants were informed about the study, and their informed consent was obtained.

### 2.2 Procedure

The study was planned in three stages. The participants were told to refrain from drinking tea or coffee and smoking before the study. HRV recording and saliva sampling began at 9.00 a.m. and lasted until 12.00 p.m. (the reason for keeping the time interval close is the circadian rhythm of the cortisol hormone). HRV recording and saliva sampling were carried out for all the participants in the first stage without listening to the sound (control). The HRV was recorded with on‐ear headphones on and eyes closed to ensure consistency with the other stages.

The second stage followed immediately after the first stage. The same sound was played as 3D sound to one group and as mono sound to the other group. HRV was recorded during the listening, and saliva samples were collected 15 min later. HRV recording was taken with eyes closed. The purpose of playing 3D sound to one group and mono sound to the other group in the study was to minimize the variation that may take place during listening depending on the sound type since we played the same sound.

**FIGURE 1:**
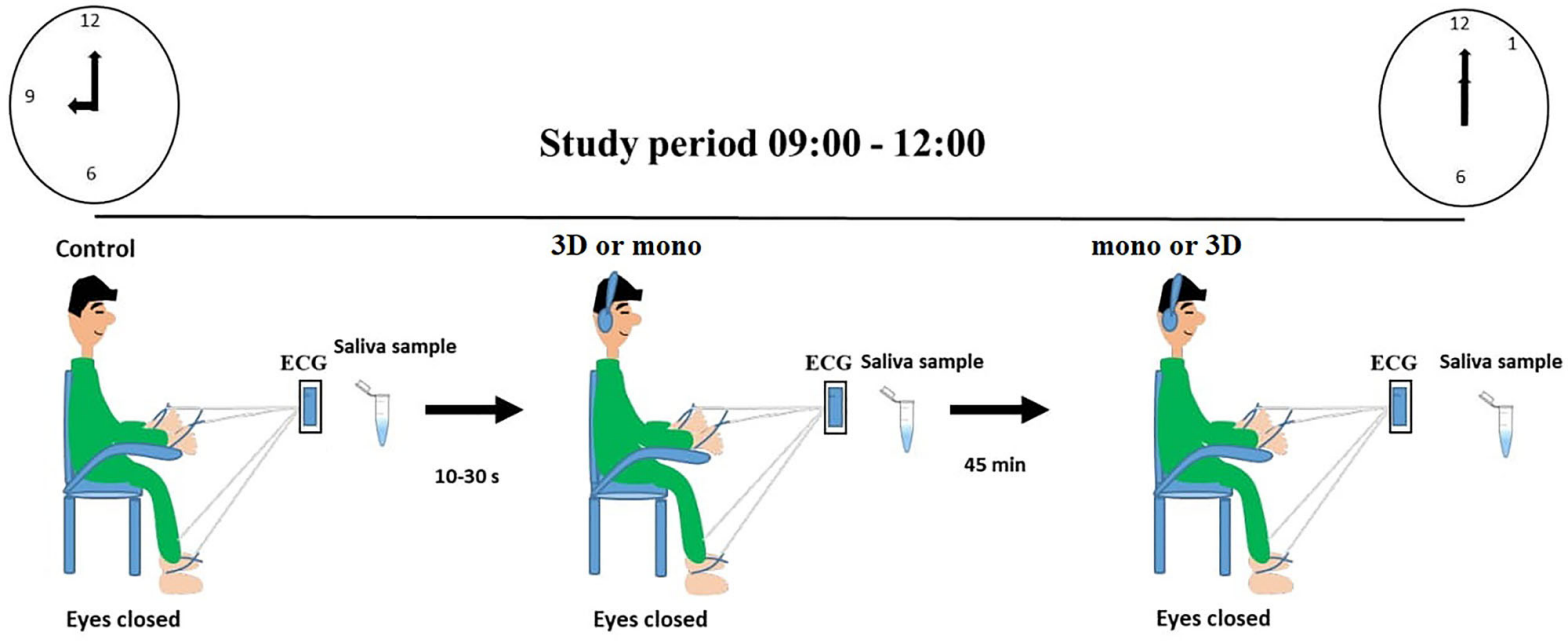
The study was planned in three stages. HRV recording and saliva sampling began at 9:00 a.m. and lasted until 12:00 p.m. In the first stage, HRV recording and saliva sampling were conducted for all participants without any practice (control). (The HRV was recorded with headphones on and eyes closed to ensure consistency with the other stages. Nothing was played.) The second stage followed immediately after the first stage. The same sound was played as a three‐dimensional sound for one group and as a mono sound for the other group. HRV was recorded during the listening, and saliva samples were collected 15 min later. HRV recording was conducted with eyes closed. The purpose of playing 3D sound for one group and mono sound for the other group was to minimize the variation that could occur during listening depending on the sound type, as the same sound was played. The third stage took place 45 min after the second stage. The participants who listened to 3D sound in the second stage listened to the same sound in mono, and the participants who listened to mono sound in the second stage listened to the sound in 3D. HRV recording was conducted during listening, and saliva samples were collected 15 min later.

The third stage took place 45 min after the second stage. The participants who listened to 3D sound in the second stage listened to the same sound in mono, and the participants who listened to mono sound in the second stage listened to the sound in 3D. HRV recording was taken during listening, and saliva samples were collected 15 min later (Figure 1).

The influence of the measurement sequence (mono first, then 3D, or 3D first, then mono) on various parameters was evaluated using the repeated measures two‐way analysis of variance. According to the results, HR and LF/HF ratios were significantly affected by the measurement order (*p* = 0.005 and 0.042, respectively), while no significant differences were observed in other parameters. These results validate the protocol we implemented.

### 2.3 The Content of the Sound Played

The sound imitating a virtual barbershop was produced by QSound Labs (https://www.qsound.com/demos/binaural‐audio.htm), and a written permit was obtained to use it in this experiment. Briefly, the record continues for 5 min, with the first 2.5 min consisting of general sounds in a barbershop, whereas the second 2.5 min is being related to haircuts with scissors and machines. The participants listened to this virtual reality environment produced by 3D sound using on‐ear headphones. At the beginning of the recording, a door opens on the right side, and someone walks next to you. The person who enters begins to inform you about the sound. Meanwhile, a second person walks in by knocking on the door on the right. While the first person pulls a chair on the left and starts playing the guitar, the last person who comes in puts a plastic bag over your head and takes it off (it creates a feeling of relief while taking it off). Afterward, while the phone rings on the left, someone washes their hands on the right. The last person to come in creates a finger‐snapping sound on the left and right. During the second 2.5 min, a haircut starts with scissors and continues with the machine, leading to experiences related to haircuts (it creates the feeling that the hair is really cut). While these scenes are going on, the sound of playing guitar continues on the left side. While the dialogues keep going, the last person to come in walks around and suddenly whispers in your ear (you can feel his breath in your ear).

### 2.4 Determining the Level of Cortisol

A competitive ELISA kit (Y Immunotek A.Ş., Malatya/Türkiye) was used for the determination of cortisol concentration in saliva samples taken from the participants (Ozgocer, Yildiz et al. 2017). The sensitivity of the test was 1 ng/mL. The measuring range was 1–1000 ng/mL. Intra‐ and inter‐assay coefficients of variation were 10.4% and 13.1% for low‐quality controls and 8.1% and 12.2% for high‐quality controls, respectively.

### 2.5 Heart Rate Variability

HRV was recorded for 5 min in a sitting position. After the HRV was recorded as a control, the other HRV was recorded during the playback of the sounds (3D and mono). The Poly‐Spectrum‐8/E device was used for HRV recording, and HRV recording (Table 1) was analyzed using the software (Neurosoft, Ivanovo, Russia).

**TABLE 1:** HRV Recording (Shaffer and Ginsberg 2017 **)**.

Time‐dependent parameters	Unit	Description
HR	(bpm)	Heart rate per minute
SDNN	(ms)	The standard deviation of the NN intervals. A criterion for medical classification of cardiac risk.
RMSSD	(ms)	The root mean square of consecutive RR interval differences. It reflects the beat‐to‐beat variance in heart rate and is the primary time domain metric used to estimate vagally‐mediated changes reflected in heart rate.
pNN50	(%)	The percentage of consecutive RR intervals that vary by more than 50 ms.
TP	(%)	The sum of the energies in the VLF, LF, and HF bands.
**Frequency‐dependent parameters**	**Unit**	**Description**
VLF	(ms^2^)	A very low frequency (VLF) band (0.0033–0.04 Hz) is associated with post‐traumatic stress disorder.
LF	(ms^2^)	A low frequency (LF) band (0.04–0.15 Hz) reflects a person's sympathetic and baroreceptor activity.
HF	(ms^2^)	A high frequency (HF) (0.15–0.40 Hz) and low HF power are associated with stress, panic, anxiety or concern, and the HF band reflects parasympathetic activity.
LF/HF	(ms^2^)	A low LF/HF ratio reflects parasympathetic dominance, whereas a high LF/HF ratio indicates that sympathetic dominance appears itself whenever we initiate fight‐or‐flight behaviors or parasympathetic withdrawal.

### 2.6 Statistical Analyses

The data was analyzed using the IBM SPSS statistical program. The Kolmogorov–Smirnov normality test was used to determine whether the data were normally distributed. Non‐normally distributed data were converted to a log_10_ scale before statistical analyses. The repeated measures ANOVA test was used to analyze the data. Bonferroni correction for multiple comparisons was used for pairwise comparisons. The data were expressed in mean ± SEM, and the value of *p* < 0.05 was considered statistically significant.

G*Power computer program was used to determine the sample size. The sample size was calculated as 28 people in total when Type 1 error (*α*) of 0.05, type 2 error (*β*) of 0.20 (Power = 0.80), effect size (*d*) of 0.25 (medium effect), non‐sphericity correction of 1, and correlation between measures of 0.5 were accepted as a result of power analysis (repeated ANOVA test) for dependent groups (three measurements).

## 3 Results

### 3.1 Effect of 3D and Mono Sound on Cortisol Levels

Cortisol level in the control group was found to be 101.3 ± 36.9 ng/mL. When the sound was played in mono, the cortisol level was found to be 47.6 ± 69 ng/mL. When the 3D feature of the sound was played, the cortisol level was found to be 75.6 ± 24.9 ng/mL. In the statistical analysis, no statistically significant difference was found between the three groups (*p* > 0.05).

### 3.2 The Effect of 3D and Mono Sound on HRV

The data were collected from the participants after three different HRV recordings for 5 min. Table 2 shows the HRV parameters and results. It was determined that there were statistically significant differences in all HRV parameters except for HF.

**TABLE 2:** HRV parameters results.

HRV parameters	Control	Mono	3D	*p* value
HR (bpm)	85.08 ± 1.82^a^	80.42 ± 1.75^b^	82.42 ± 1.68^b^	0.000*
SDNN (ms)	41.96 ± 3.03^a^	44 ± 3.21^ab^	48.07 ± 3.34^b^	0.004*
RMSSD (ms)	30.65 ± 3.05^a^	35.54 ± 3.91^ab^	35.85 ± 3.65^b^	0.002*
pNN50 (%)	9.48 ± 1.99^a^	13.25 ± 2.65^ab^	11.58 ± 2.11^b^	0.025*
TP (ms^2^)	2364 ± 420^a^	2376 ± 337^a^	3070 ± 495^b^	0.005*
VLF (ms^2^)	1017 ± 194^a^	861 ± 115^a^	1354 ± 286^b^	0.010*
LF (ms^2^)	771 ± 173 ^a^	802 ± 124 ^a^	1055 ± 160 ^b^	0.000*
HF (ms^2^)	576 ± 109 ^a^	713 ± 164 ^a^	669 ± 128 ^a^	0.627
LF/HF (ms^2^)	2.04 ± 0.21^a^	2.44 ± 0.27^ab^	2.79 ± 0.38 ^b^	0.021*

*Note*: The letters “a” and “b” are used to describe differences between measurements. Different letters in a row describe statistically significant differences. Overall, statistical differences have been marked by asterisks and as bold font.

In the statistical analysis, only the HR parameter was the highest in the control group, while pNN50 and HF were higher in the mono group. All other parameters were the highest in the 3D group.

**FIGURE 2:**
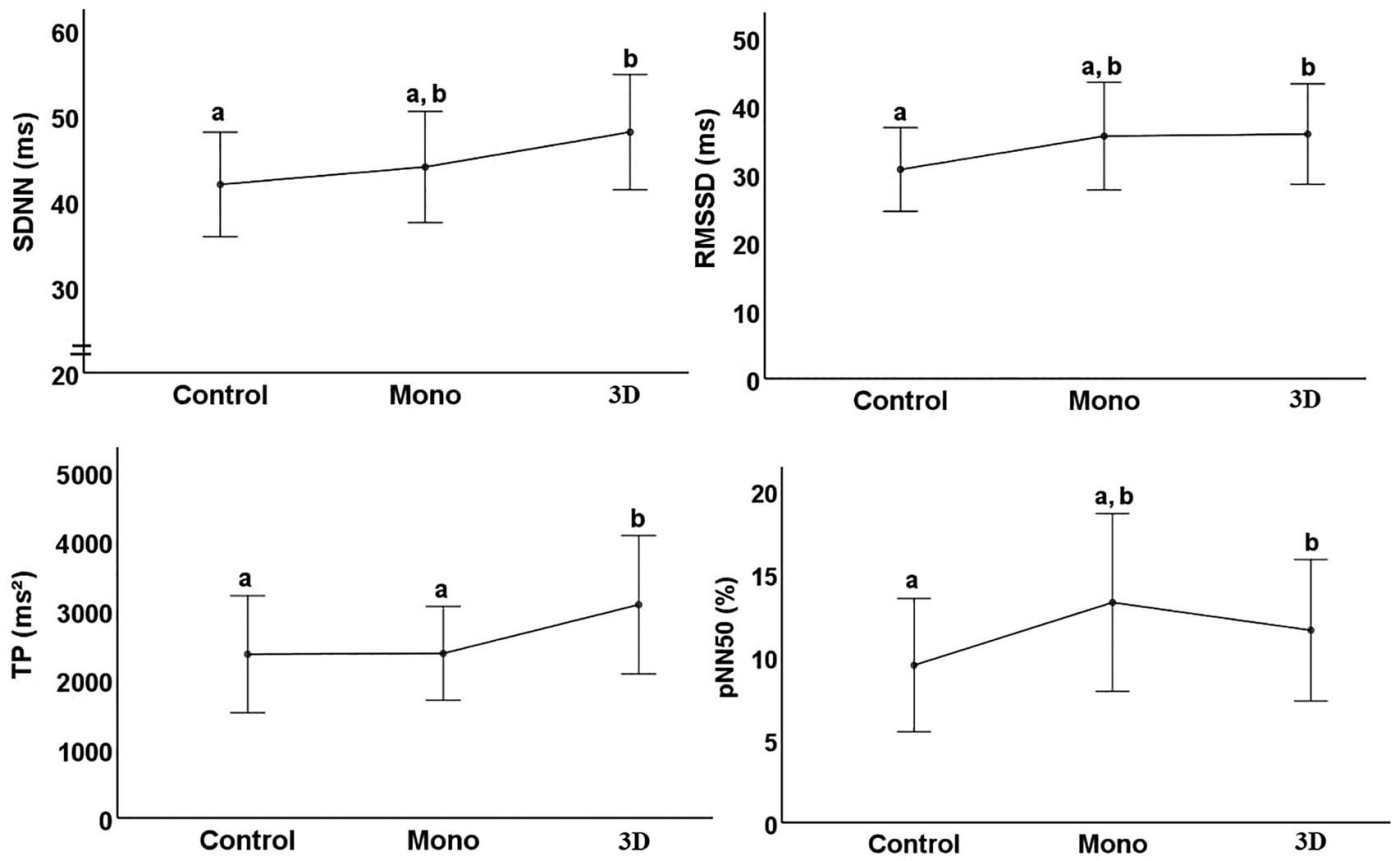
HRV results between groups in frequency‐dependent parameters.

In frequency‐dependent parameters, when the groups were compared within themselves, the HR level in the control group was higher than the HR level in mono and 3D, with a statistically significant difference. There was no statistically significant difference between mono and 3D. When SDNN, RMSD, and pNN50 parameters were examined, the result in 3D was significantly higher than the control group. There was no statistically significant difference between the mono group and both the 3D and control groups. In the TP parameter, the 3D group was statistically significantly higher than the control and mono groups. There was no significant difference between the control and mono groups (Figure 2).

**FIGURE 3:**
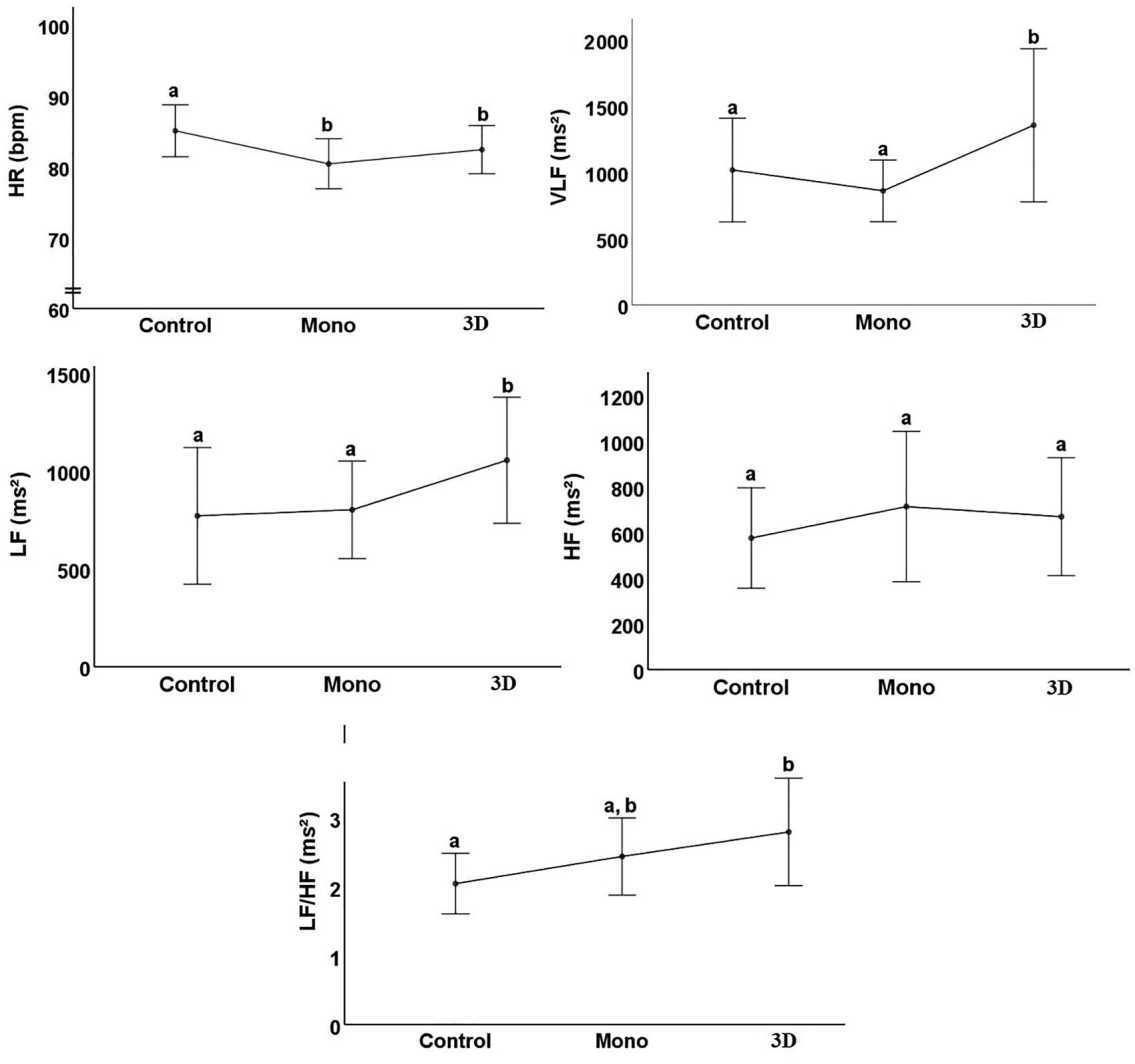
HRV results between groups in time‐dependent parameters.

When the groups were compared within themselves based on time‐dependent parameters, there was no statistically significant difference between the three groups in the HF parameter. The 3D group was significantly higher in VLF and LF parameters than the control and mono groups. There was no significant difference in the control and mono groups. In the LF/HF parameter, the result in 3D was significantly higher than in the control group. There was no statistically significant difference between the mono group and both the 3D and control groups (Figure 3).

### 3.3 Comparison of the Data Within 3D Sound

The first half of the 3D sound was about the general situation in a barbershop, whereas the second half was related to virtual haircuts. Therefore, we made an additional attempt to compare these stages. When the difference between the first 2.5 min and the last 2.5 min of 3D sound was analyzed, it was found that there was a statistically significant increase in HR and LF/HF in the last 2.5 min, while no significant difference was observed in SDNN, RMSSD, pNN50, TP, VLF, LF, and HF parameters (Table 3).

**TABLE 3:** Difference between the first 2.5 min and the last 2.5 min in 3D sound.

HRV Parameters	first 2.5 min	Last 2.5 min	*p* value
HR (bpm)	83.38 ± 1.84	84.68 ± 1.79	0.005*
SDNN (ms)	44.55 ± 3.44	43.36 ± 3.36	0.933
RMSSD (ms)	32.92 ± 3.54	32 ± 3.48	0.496
pNN50 (%)	13.19 ± 2.63	12.09 ± 2.44	0.102
TP (ms^2^)	2301 ± 359	2180 ± 311	0.984
VLF (ms^2^)	792 ± 144	753 ± 101	0.952
LF (ms^2^)	887 ± 176	901 ± 142	0.667
HF (ms^2^)	622 ± 149	526 ± 115	0.220
LF/HF (ms^2^)	2.88 ± 0.44	3.45 ± 0.57	0.048*

*Note*: Overall statistical differences have been marke7d by asterisks and as bold font.

## 4 Discussion

The current study showed that listening to a 3D virtual reality sound imitating a real‐life environment taking place in a barbershop affected the ANS but not the HPA axis, suggesting that the sympathetic stress management system of the human body is sensitive to virtual 3D sound environments. This may have practical implications in the digitalizing world as an increasing number of people are trying to adopt segregated individual lifestyles. From that point of view, the current study suggests that virtual reality‐created 3D sound environments enriched with real‐world dialogues may help people living in lonely conditions by keeping up the activity of the autonomous nervous system. Moreover, as the HPA system was not different between the phases, it might be speculated that the effect of 3D VR sounds on ANS, but not on HPA, is immediate. Rather, to have an effect on HPA, a longer duration and higher intensity of disturbing sounds might probably be required. Overall, the findings of the study have been discussed below by taking into account current literature knowledge.

### 4.1 As Measured by HRV, 3D VR Sound Had a Profound Impact on ANS

HRV technique assesses autonomic nervous activity by analyzing cardiac inter‐beat intervals. Among the parameters obtained from HRV, the sympathovagal balance of the ANS is reported to be presented by the LF/HF parameter. In the current study, the LF/HF parameter increased significantly compared to the control measurement. This suggests that sympathetic activity, which governs the “fight or flight” mechanism in the body, is quickly activated by the 3D VR technology. Thus, the LF/HF parameter of HRV appears to be quite sensitive for these types of studies. Furthermore, it seems that the sympathovagal system is more closely associated with immediate events, as it is observed during the listening period.

In the current study, 3D VR sound not only increased LF/HF but also increased TP and SDNN parameters. TP and SDNN measures represent frequency‐ and time‐domain parameters of HRV and are associated with the total “variability” of heart rate. HRV has also been closely associated with the general status of health. Increased HRV has been associated with decreased depression rate (Taylor 2010; Kumar et al. 2024), coronary heart disease, heart failure (Baig et al. 2022), kidney failure (Ranpuria et al. 2008), and hypertension (He et al. 2024). There are numerous studies that have examined the effect of sound on HRV (Dousty et al. 2011; Pérez Lloret et al. 2014; Lee et al. 2010; da Silva and Backs 2015; Orini et al. 2010). Although not equivocal, these studies contributed critical information; they have generally focused on noise, music, and sounds of different frequencies on HRV. No study has examined the 3D VR sound imitating a real‐world environment on HRV. The current study adds that apart from noise or selected frequencies of sounds, real‐life sound content (barbershop) affects HRV, especially if it is created by 3D VR. Although mono sound was also able to increase HRV parameters, its magnitude was not as high as 3D VR technology. This suggests that our brain is especially reactive to the content of the sound if it is more realistic. Interestingly, the current study also shows that these types of effects on HRV parameters are observed even if the visual component of the VR technology is absent.

The current study also showed that 3D VR sound increased RMSSD, which is generally attributed to increased vagal activity. There is a study reporting an increase in RMSSD in musical sounds played in 3D (Roy et al. 2012). This RMSSD increase may be related to increased attention in people listening to 3D sounds because increased attention can stimulate parasympathetic activity (Barber et al. 2020). However, there was no difference in the HF parameter. When the results were examined again, it was seen that there was an increase in HF in mono and 3D sounds, but this increase was not significant. However, this increase may show a significant increase for the RMSSD parameter reflecting vagal activity. As discussed above, 3D VR sound increased the LF/HF parameter, a marker for increased sympathovagal activity. This conflicting finding might be related to the complexity of the ANS system, the HRV technique, or the sound listened to. It is not impossible to simultaneously do two tasks related to the sympathetic and parasympathetic systems. For example, a person might watch a horror film (sympathetic activity) while eating/drinking something (parasympathetic activity). Additionally, the HRV technique is a very sensitive and promising technique representing valuable information but its interpretation still needs further improvement. Lastly, the sound listened to was very complex, and we made an effort to test whether the first half of the sound (which was related to the general environment related to a barbershop) was different from the second half of the sound (which was related to a haircut by scissors and machine). The results showed that, depending on the perception of sound‐related events, HRV parameters may also differ within the phases. This suggests that specific sound‐associated events might increase the sympathetic or parasympathetic branches of the ANS. As a result, HRV appears to be a very dynamic responder technique towards sound‐related VR environments.

### 4.2 3D VR Sound Did Not Affect HPA Axes

Cortisol is the end product of the HPA axis and an integral parameter of stress studies. In this study, we hypothesized that 3D VR sound would reduce cortisol levels but no significant difference was observed in cortisol levels. There might be various explanations for this. Firstly, probably the participants did not have measurable stress conditions that could be reduced by a 3D VR sound environment. Moreover, the diurnal nature of cortisol release could not be the cause of this situation, as our study employed a crossover design. Additionally, by using participants as their own controls, interindividual variability was minimized through paired sampling. Secondly, the sound stimuli used in this study may have been below the threshold in terms of intensity and duration of application. Moreover, the sound environment listened to was not sufficiently disturbing to activate the stress‐inducing threshold of HPA. Participants reported that 3D VR sound was more enjoyable, while mono sound was perceived as more boring. However, the perceived boredom of mono sound did not reach a level that could influence the HPA axis. The audio content included potentially stress‐inducing sounds, such as haircuts and whispering in the ear, but these did not impact the HPA axis. When considering all these findings, it might be suggested that the HPA axis is less sensitive to momentary stress and is more reflective of long‐term stress. A recent study we conducted on earthquake victims reported that the HPA axis might be involved in long‐term stress, whereas the ANS appeared to be more responsive to momentary stress (Yilmaz et al. 2024). These findings lend support for the results obtained in the current study.

There are many studies that have examined the effect of sound on cortisol levels (Hébert et al. 2005; Tan et al. 2014; Pal et al. 2022; Helsing et al. 2016; Al‐Shargie et al. 2021). However, the literature review revealed no study in which the effects of 3D and mono sound on individuals were examined. The studies in the literature showed that they have generally focused on the effects of noise, music, and sounds of different frequencies. It has been reported that listening to music while playing games increases cortisol levels more than silence (Hébert et al. 2005). On the other hand, Tan et al. (2014) found no difference in salivary cortisol levels between music and silence. It has also been reported that noise augments cortisol release (Pal et al. 2022), and listening to music lowers cortisol levels (Helsing et al. 2016). Al‐Shargie et al. (2021) reported that cortisol levels might fall in people who listened to different frequencies with binaural beats. The present study adds that a 3D VR mimicking a normal life environment does not affect cortisol levels and that there is no difference between mono and 3D sounds.

## 5 Strengths and Limitations of the Study

In the current study, paired sampling and crossing half of the participants between the mono and 3D VR sound eliminated interindividual error. Unfortunately, we were unable to make entropy analyses like the refined composite multiscale entropy (RCMSE) parameter, because the software of the HRV device we used was not suitable for this analysis.

## 6 Conclusion

The current study analyzed the effect of 3D VR sound imitating a real‐life environment on stress axes and found out that ANS, but not HPA, was responsive to the auditory perception. Firstly, the findings suggest that real‐world environments might be imitated by 3D VR sounds, as revealed by the increased activity of ANS. The results also suggest that auditory input was not of sufficient duration and strength to activate the HPA axis. Interestingly, the HRV technique dynamically responded to the stages of 3D VR sound environments without a need for the visual component of the VR technology. Therefore, it seems that HRV is a highly sensitive technique for use in these types of studies imitating real‐life conditions.

## Author Contributions

**Yücehan Yilmaz**: writing–original draft, resources, methodology, conceptualization, writing–review and editing, investigation. **Faruk Dişli**: validation, formal analysis, writing–review and editing. **Sedat Yildiz**: writing–review and editing, writing–original draft, methodology, conceptualization.

## Conflicts of Interest

The authors declare no conflicts of interest.

### Peer Review

The peer review history for this article is available at https://publons.com/publon/10.1002/brb3.70418

## Data Availability

The data that support the findings of this study are available on request from the corresponding author. The data are not publicly available due to privacy or ethical restrictions.
